# The complex nest architecture of the Ponerinae ant *Odontomachus chelifer*

**DOI:** 10.1371/journal.pone.0189896

**Published:** 2018-01-03

**Authors:** Ingrid de Carvalho Guimarães, Márlon César Pereira, Nathan Rodrigues Batista, Candida Anitta Pereira Rodrigues, William Fernando Antonialli

**Affiliations:** 1 Laboratório de Ecologia Comportamental, Centro de Estudos em Recursos Naturais, Universidade Estadual de Mato Grosso do Sul, Dourados, Mato Grosso do Sul, Brazil; 2 Programa de Pós-graduação em Recursos Naturais, Universidade Estadual de Mato Grosso do Sul, Dourados, Mato Grosso do Sul, Brazil; 3 Programa de Pós-graduação em Entomologia e Conservação da Biodiversidade, Universidade Federal da Grande Dourados, Dourados, Mato Grosso do Sul, Brazil; Hungarian Academy of Sciences, HUNGARY

## Abstract

In social insects, nests are very important structures built to provide a protected microhabitat for immature development and food storage and are the places where most interactions between all members of a colony occur. Considering that nest architecture is an important behavioural trait that can clarify essential points of the social level of the species, here we describe the architectural model of the Ponerinae ant *Odontomachus chelifer*. Five subterranean nests were excavated; one of them filled with liquid cement for extraction of casts of chambers, shafts and tunnels. All nests were found in a woodland area, with Dystrophic Red Latosol soil, associated with roots of large trees and, differently from the pattern currently described for this subfamily, presented a complex structure with multiple entrances and more than one vertical shaft connected by tunnels to relatively horizontal chambers. The number of chambers varied from 24 to 77, with mean volume ranging from 200.09 cm^3^ to 363.79 cm^3^, and maximum depth of 134 cm. Worker population varied between 304 and 864 individuals with on average 8.28 cm^2^ of area per worker. All nests had at least one Hall, which is a relatively larger chamber serving as a distribution centre of the nest, and to our knowledge, there is no record of Ponerinae species building similar structure. All nests had chambers "paved" with pieces of decaying plant material and on the floor of some of them, we found a fungus whose identification and function are being investigated. Thus, our findings provide evidence to suggest that nests of *O*. *chelifer* can be considered complex, due to the great number and organization of chambers, shafts and connections, compared to those currently described for Ponerinae species.

## Introduction

Nests are the places where most interactions between members of colonies of social insects occur and their main function is to provide a protected microhabitat for immature development and food storage [1]. Maintaining the colonies’ cohesion requires a constant flow of information between members of the colony, including during construction and expansion of nests which, in this regard, are not just a result of ant interactions but also mediators of this transmission of information through a form of indirect communication called stigmergy [1,2,3]. Thus, building these structures requires that workers work together performing different tasks to complete a sub-task [4].

Ant nests consist of two main elements, vertical shafts connected by tunnels to relatively horizontal chambers [5]. The variation in shape, size and spatial distribution of these elements results in different architectural models [5,6,7]. In general, nests provide a range of microclimates and temperatures suitable for brood rearing [5,7]. By not having cells, unlike nests of wasps and bees, ant nests are places where the interactions between immature-immature and adults-immature happen freely [1]. In addition, because they are carefully structured to provide a safe and stable microhabitat, other animals are often found using them as places for feeding, breeding, or shelter [8,9,10,11,12,13].

There are few studies that describe nest architecture of Ponerinae ants, which generally present nests with small number of chambers, without branches, often built by modifying and increasing natural cavities [10,14,15]. As is the case of the species: *Dinoponera quadriceps* Kempf 1971 which build nests near or associated with trees, branches or fallen trunks, with few chambers and maximum depth of 1.2 m [16], and sometimes occupy empty nests of leaf-cutting ants [17]; *Dinoponera lucida* Emery 1901 whose nests present chambers following the axis of tree roots on litterfall and reach maximum depth of 35 cm [18]; *Dinoponera australis* Emery 1901 featuring nests with few chambers arranged along a helicoidal axis [18]; *Pachycondyla striata* Smith 1858 whose nests are poorly elaborated with chambers and tunnels near the ground surface [13], among others. On the other hand, some species of this subfamily build more complex nests, such as *Harpegnathos saltator* Jerdon 1851, which uses a papery material to build their nests and have the upper chamber protected by a particular vaulted roof on the outside of which there is an intervening space that isolates the chambers from the ground conferring protection against flooding [15].

Species of the genus *Odontomachus* Latreille 1804 have several forms of nesting. *Odontomachus hastatus* Fabricius 1804 are arboreal ants and build their nests occupying spaces between roots of epiphytic bromeliads; therefore, these nests do not have a system of chambers and tunnels [19]. *Odontomachus bauri* Emery 1892 uses natural refuges, such as spaces under rocks and hollow trunks to house the colony, thus building superficial nests [14,20]. Nests of *Odontomachus brunneus* Patton 1894, on the other hand, consist of a single vertical shaft connecting simple relatively horizontal chambers, ranging from 18 to 184 cm deep [6]. Therefore, previous studies have shown that ants of this genus, as other ponerines, build nests with few chambers and connections and relatively superficial.

Considering that nest architecture is an important behavioural trait and can clarify relevant points of the social level of the species and that there are still few studies on building patterns of subterranean ant nests, especially of Ponerinae species, here we describe the nest architecture of *Odontomachus chelifer* Latreille 1802 and demonstrate that they are more complex than the ones of the majority of Ponerinae ants currently described.

## Materials and methods

We excavated 5 subterranean nests of *O*. *chelifer* from April 2016 to March 2017, in the municipality of Dourados-MS (22°13’16”S; 54°48’20”W). One of them was filled with liquid cement for extraction of casts of the chambers, shafts and tunnels. All colonies were nested in a woodland area with Dystrophic Red Latosol soil [21]. Field permission was granted by Maria Aparecida Betoni responsible for the land where the study was conducted. Species identification was confirmed by Prof. Dr. Jacques H. C. Delabie of Comissão Executiva do Plano da Lavoura Cacaueira–CEPLAC, Centro de Pesquisa do Cacau–CEPEC, Laboratório de Mirmecologia of Universidade Estadual de Santa Cruz–UESC. The field studies did not involve endangered or protected species.

The subterranean nests were excavated according to the methodology proposed by [9]. Initially, we excavated a circular trench of approximately 100 cm deep around the entrance orifices at a distance of about 50 cm radius, so that the nest was contained in a cylinder of soil. After we isolated the nest, the chambers were excavated individually by vertically slicing this cylinder. Every time we found a chamber or tunnel, the soil was sliced very carefully in order not to perturb the architecture. The chambers were cleaned with a fine brush and measured. The structures were preserved until we had excavated, measured and drawn all elements of the shaft before beginning the next one. Whenever we found tree roots, we made sure to preserve the architecture by cutting them very carefully without making sudden and jerky movements.

Whenever possible, from each nest we recorded the following data: number of individuals; maximum depth (MD); number of shafts (NS), each vertical system connecting chambers one relatively below the other was called “shaft”; number of chambers (NC); number of entrance orifices (NE), diameter in length (the longer two edges) and width (the shorter two edges) of each entrance orifice [9,22]. In addition to depth (from ground surface to chamber’s roof), length (the longer two edges), width (the shorter two edges) and height (from chamber’s floor to roof) of each chamber [9,22]. These data were used to calculate the total volume of chambers (TVC), mean volume of chambers (MVC), total area of chambers (TAC), mean area of chambers (MAC) and area per worker (AW). All nests were drawn during excavations and posteriorly schematized based on the drawings and data recorded. Other supplementary information were recorded when it was appropriate, such as presence of other animals, plant remains and use of chambers for immature allocation or garbage storage.

We also performed Pearson’s Correlation tests to assess whether there is correlation between total volume of chambers and number of chambers; total area of chambers and number of chambers; total volume of chambers and number of workers and total area of chambers and number of workers. In order to put the area per worker into the perspective of ant size, ants collected from the nests were measured for body length with and without mandibles, head width between the eyes, gaster width at the largest part, thorax width at the largest part and lateral extension of femur of the second pair of legs. These data were used to calculate the area of the dorsal silhouette of ants with and without the legs modified from [6].

### Making 3D cast in cement

To obtain the 3D cast, we choose a relatively small nest, with few entrance orifices (n = 3) and close to a young tree, so there were fewer roots facilitating the pouring of cement. We poured a mixture of 16 kg of cement for 80 l of water with addition of two measures of lime substitute additive according to the manufacturer’s guidance on all entrances until the content overflowed, indicating that all volume had been filled, following the methodology used by [23,24,25]. After 15 days, the nest was dry enough to be excavated by the same method described above. After excavation, casts of chambers, shafts and tunnels were cleaned and carefully reconstructed using epoxy to cement the pieces together. We also recorded the same data described for the excavated nests, excepted for the number of individuals and area per worker since it was not possible to collect the ants from this nest before pouring the cement.

## Results

All nests were found in a shaded area of woodland, usually following the axes and shapes of roots of large trees and, differently from other species of the subfamily Ponerinae, they presented a complex structure with more than one vertical shaft connected by tunnels to relatively horizontal chambers. The number of chambers ranged from 24 to 77, with mean volume ranging from 200.09 cm^3^ to 363.79 cm^3^ and total volume between 4700.05 cm^3^ and 20 676.08 cm^3^. The mean depth was 97.70 ± 21.35 cm, and maximum 134 cm (Table 1). The population of workers varied between 304 and 864 individuals with area per worker ranging from 6.41 cm^2^ to 10.22 cm^2^, i.e. an average of 8.28 ± 1.75 cm^2^ per worker (Table 1). The dorsal silhouette of workers of *O*. *chelifer* measures about 9.9 ± 1.03 mm^2^ without legs and mandibles, and about 53.85 ± 5.51 mm^2^ with the extension of the lateral femurs included. Therefore, the dorsal silhouette without legs occupies an average of 1.21 ± 0.12% of the area available per worker, and with legs 6.50 ± 0.67%.

**Table 1:** Population and dimensional data of nests of *Odontomachus chelifer*.

**Nest**	**Date**	**Number of individuals**				**NE**	**NS**
		**Eggs**	**Larvae**	**Pupae**	**Adults**		
1	19/04/2016	Present	0	55	304	10	5
2	30/04/2016	Present	25	80	522	6	6
3	09/09/2016	Present	61	9	305	4	4
4	14/09/2016	Present	130	2	864	9	8
5	28/03/2017	-	-	-	-	3	4
**Nest**	**TVC**	**MVC**	**TAC**	**MAC**	**AW**	**MD**	**NC**
1	4700.05	204.35 ± 201.36	1949.73	84.77 ± 57.17	6.41	87.00	24
2	16 370.48	363.79 ± 558.83	5337.00	118.60 ± 146.15	10.22	80.50	45
3	5402.50	200.09 ± 179.30	2211.00	81.89 ± 59.71	7.25	99.00	27
4	20 676.08	268.52 ± 279.89	7962.50	103.41 ± 96.52	9.22	134.00	77
5	9089.25	201.98 ± 226.51	4369.00	97.09 ± 88.00	-	88.00	46

NE = Number of entrance orifices; NS = Number of shafts; TVC = Total volume of chambers (cm^3^); MVC = Mean volume of chambers (cm^3^); TAC = Total area of chambers (cm^2^); MAC = Mean area of chambers (cm^2^); AW = Area per worker (cm^2^); MD = Maximum depth (cm); NC = Number of chambers. Mean values followed by standard deviations.—Not acquired data.

**Fig 1:**
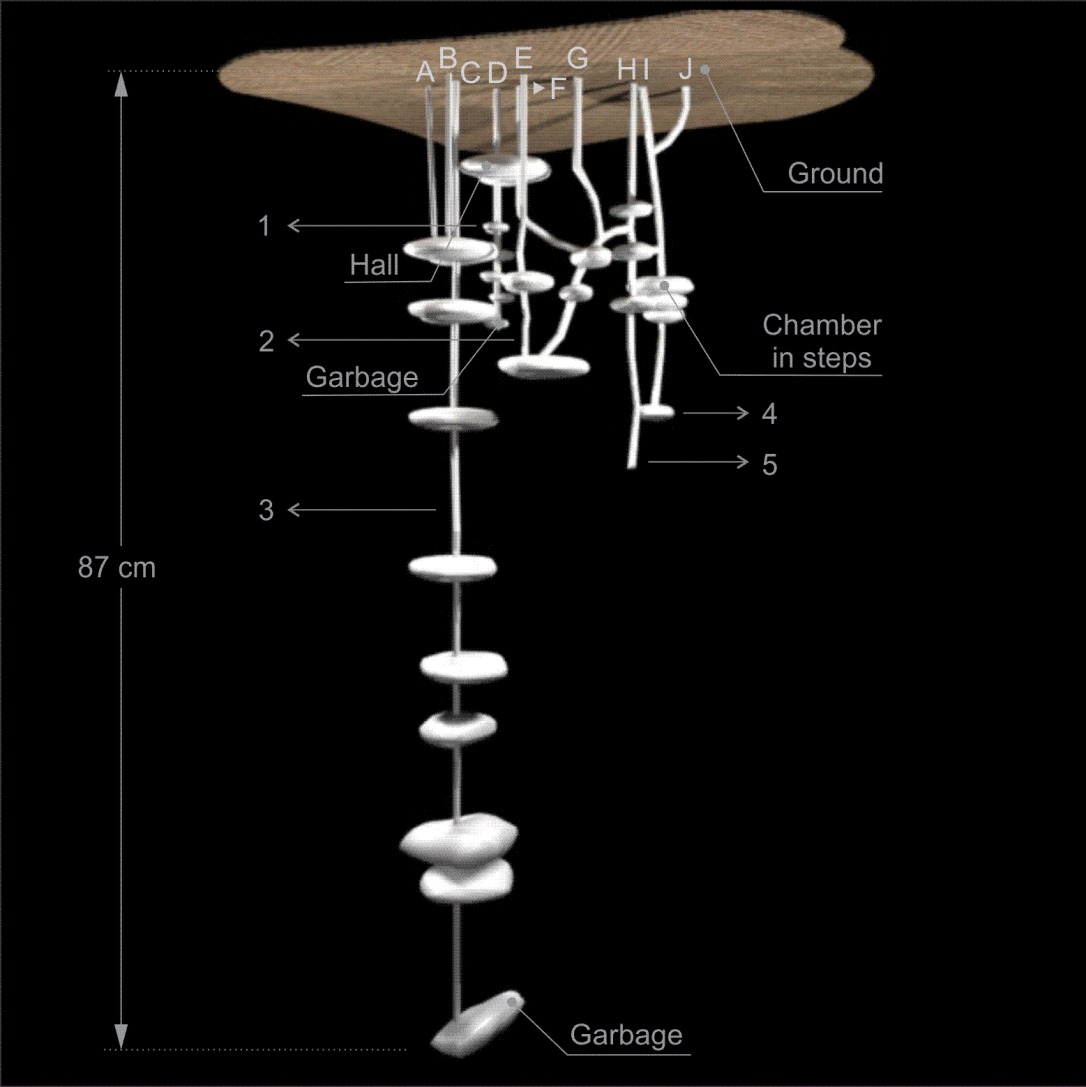
Scheme of nest 1 of *Odontomachus chelifer*. A to J = entrance orifices of the nest. 1 to 5 = Shafts of the nest. Maximum depth indicated on the major shaft.

**Fig 2:**
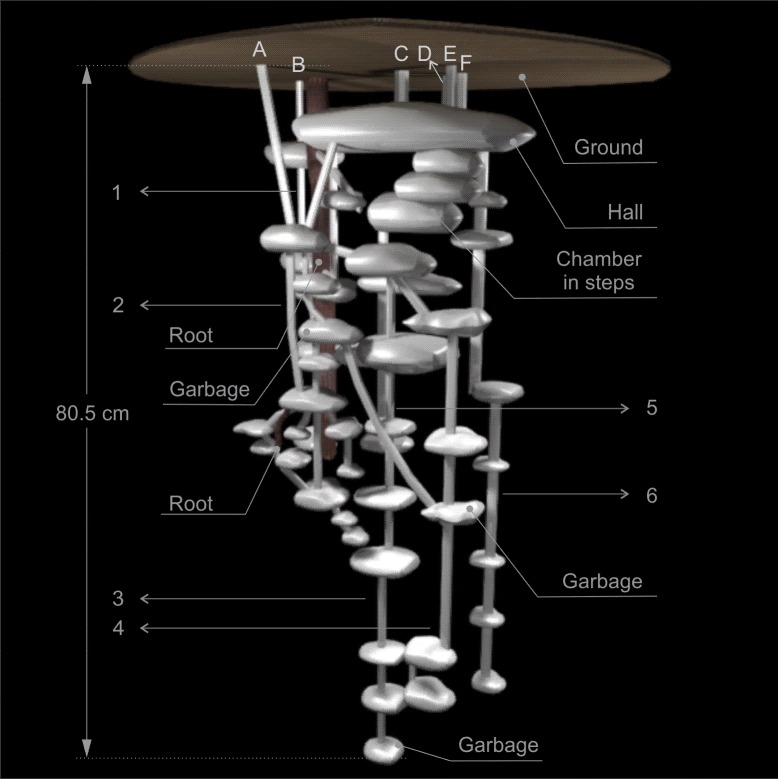
Scheme of Nest 2 of *Odontomachus chelifer*. A to F = entrance orifices of the nest. 1 to 6 = Shafts of the nest. Maximum depth indicated on the major shaft.

**Fig 3:**
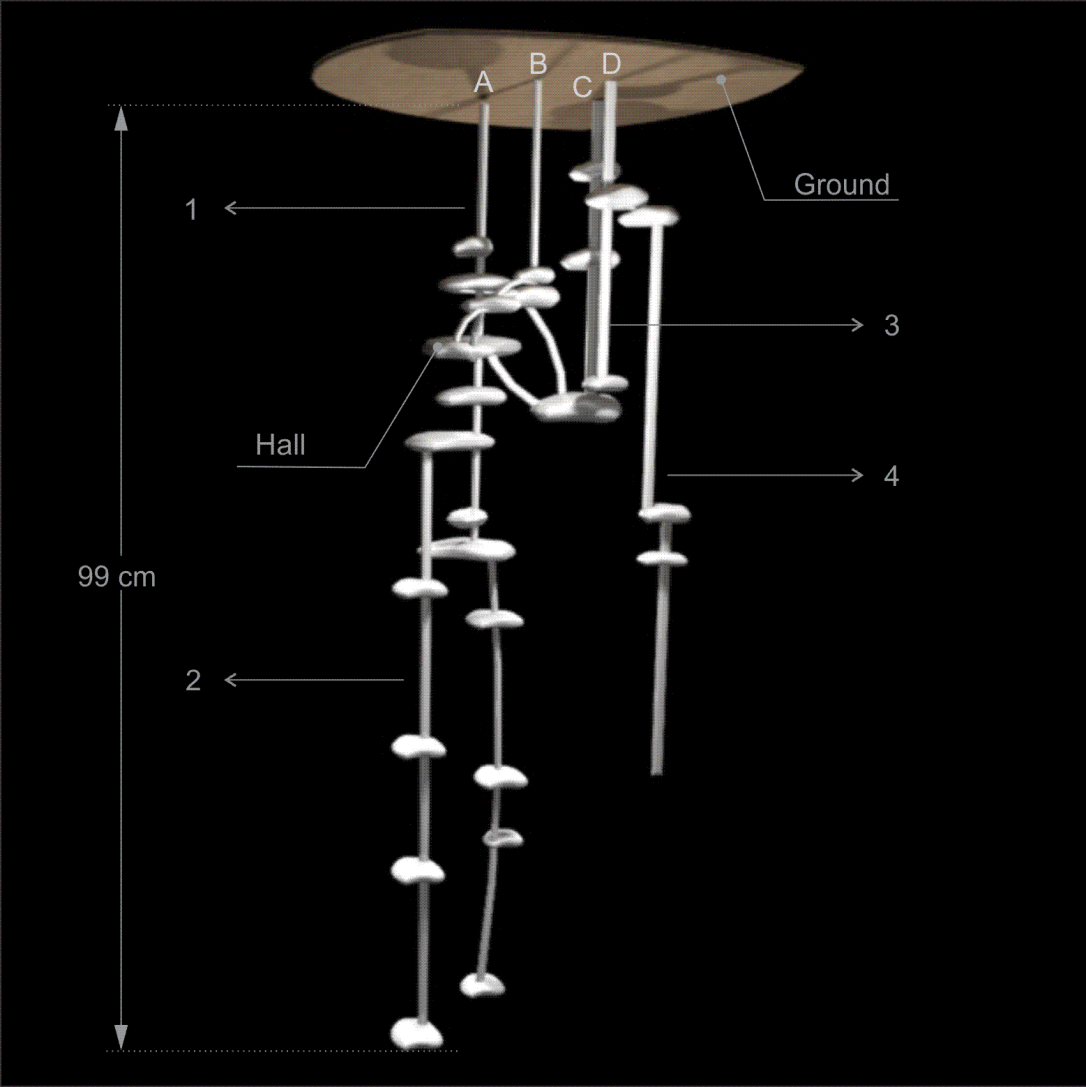
Scheme of Nest 3 of *Odontomachus chelifer*. A to D = entrance orifices of the nest. 1 to 4 = Shafts of the nest. Maximum depth indicated on the major shaft.

**Fig 4:**
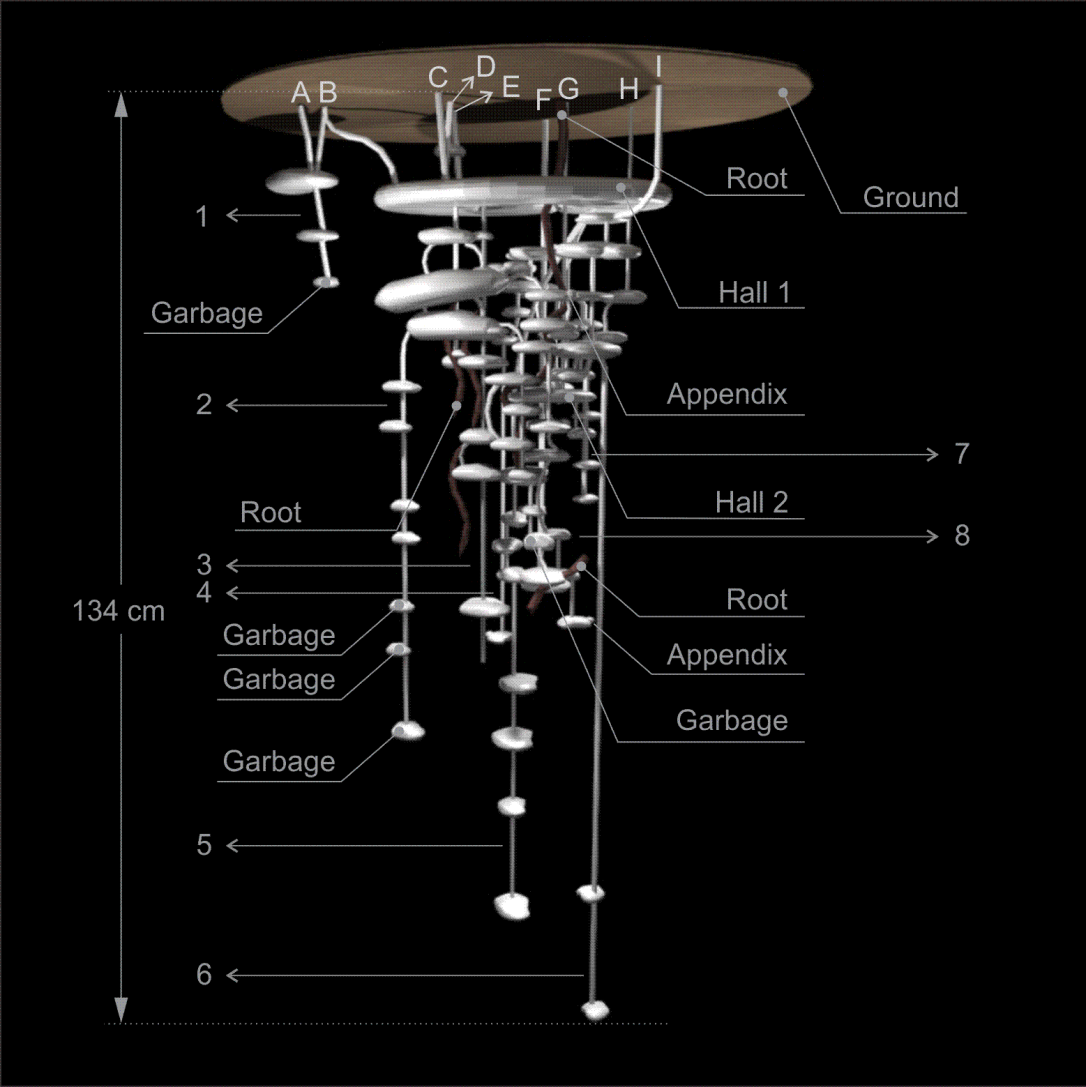
Scheme of Nest 4 of *Odontomachus chelifer*. A to I = entrance orifices of the nest. 1 to 8 = Shafts of the nest. Maximum depth indicated on the major shaft.

**Fig 5:**
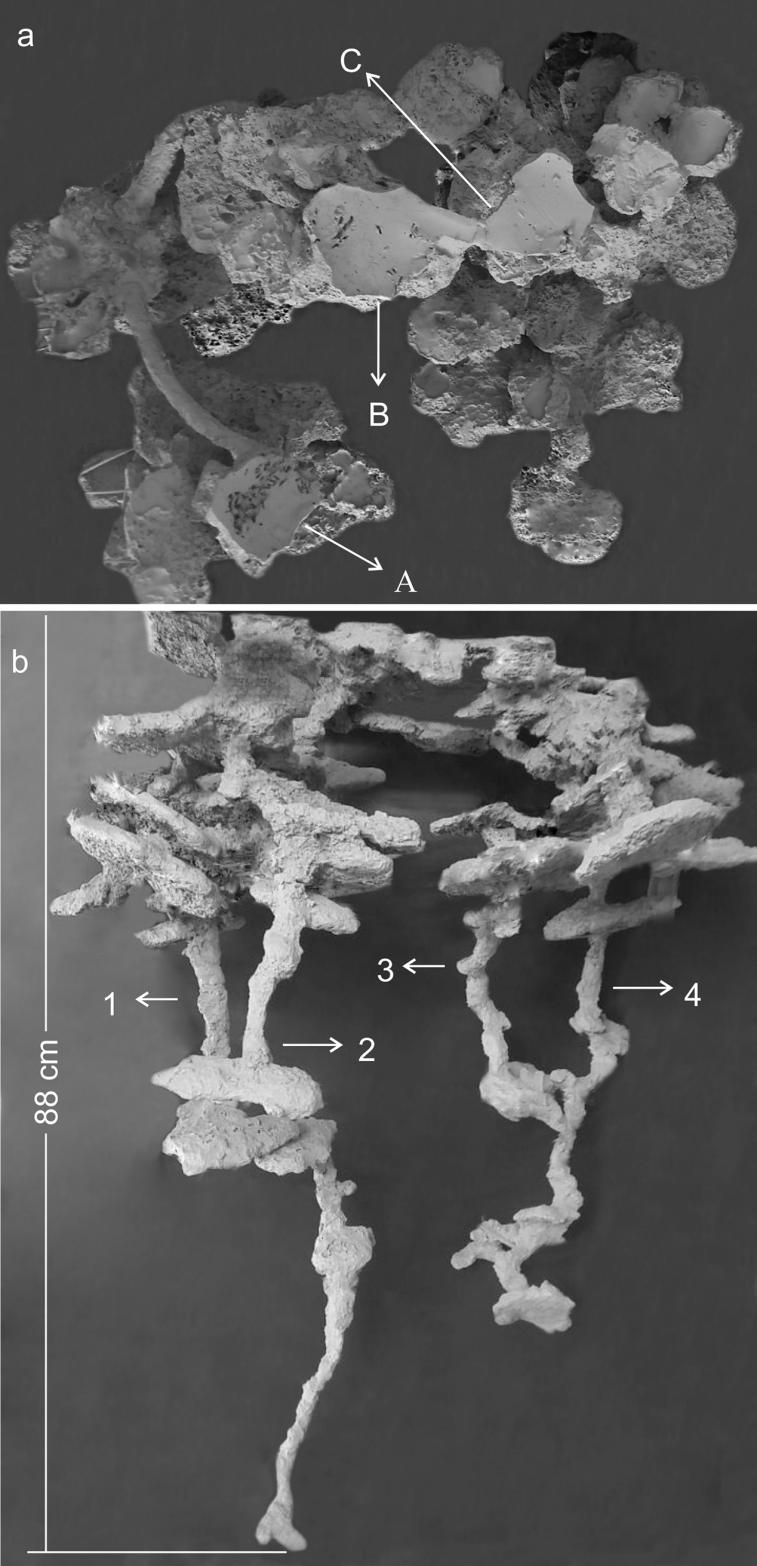
Cement cast of a relatively small *Odontomachus chelifer* nest. (a) = viewed from above. A to C = entrance orifices of the nest. (b) = Lateral view of the same nest showing the top-heavy distribution and the flattened appearance of chambers. 1 to 4 = Shafts of the nest. Maximum depth indicated on the major shaft.

Nests of this species are composed of multiple entrances and exits, ranging from 3 to 10, of various shapes, covered with litterfall material and each one usually gave access to a system of chambers, shafts and tunnels, which were usually connected underground (Figs 1–5). The entrance and exit orifices had on average 5.5 cm wide. The number of shafts ranged from 4 to 8 (Figs 1–5).

All nests had at least one relatively larger chamber with mean area of 415.21 cm^2^, where there were several tunnels connecting chambers both on the same shaft as in different shafts, serving as a distribution centre of the nest. These chambers were called "Hall" and were located on the upper or middle portion of the nest (Figs 1–4). In nest 1 two entrance orifices were connected by two tunnels to the Hall which was connected by another tunnel to other two entrances (Fig 1). In nest 2 four entrances were linked directly to the Hall, which in this case was the most superficial chamber, and the other two entrances were connected to this chamber by subterranean tunnels (Fig 2). In nest 3, differently from the previous ones, no entrance was directly linked to the Hall, which was on the upper portion of the nest below other smaller chambers; however it was connected to three entrances by tunnels and chambers, and gave access to the deepest shafts of the nest (Fig 3). Nest 4 had two major chambers called Hall 1 and Hall 2 (Fig 4). Four entrance orifices were connected by tunnels to Hall 1, which was linked to Hall 2 by another tunnel. The first Hall was located on the upper portion of the nest while the second was a bit below in the middle portion, and both gave access to several shafts of the nest (Fig 4).

**Fig 6:**
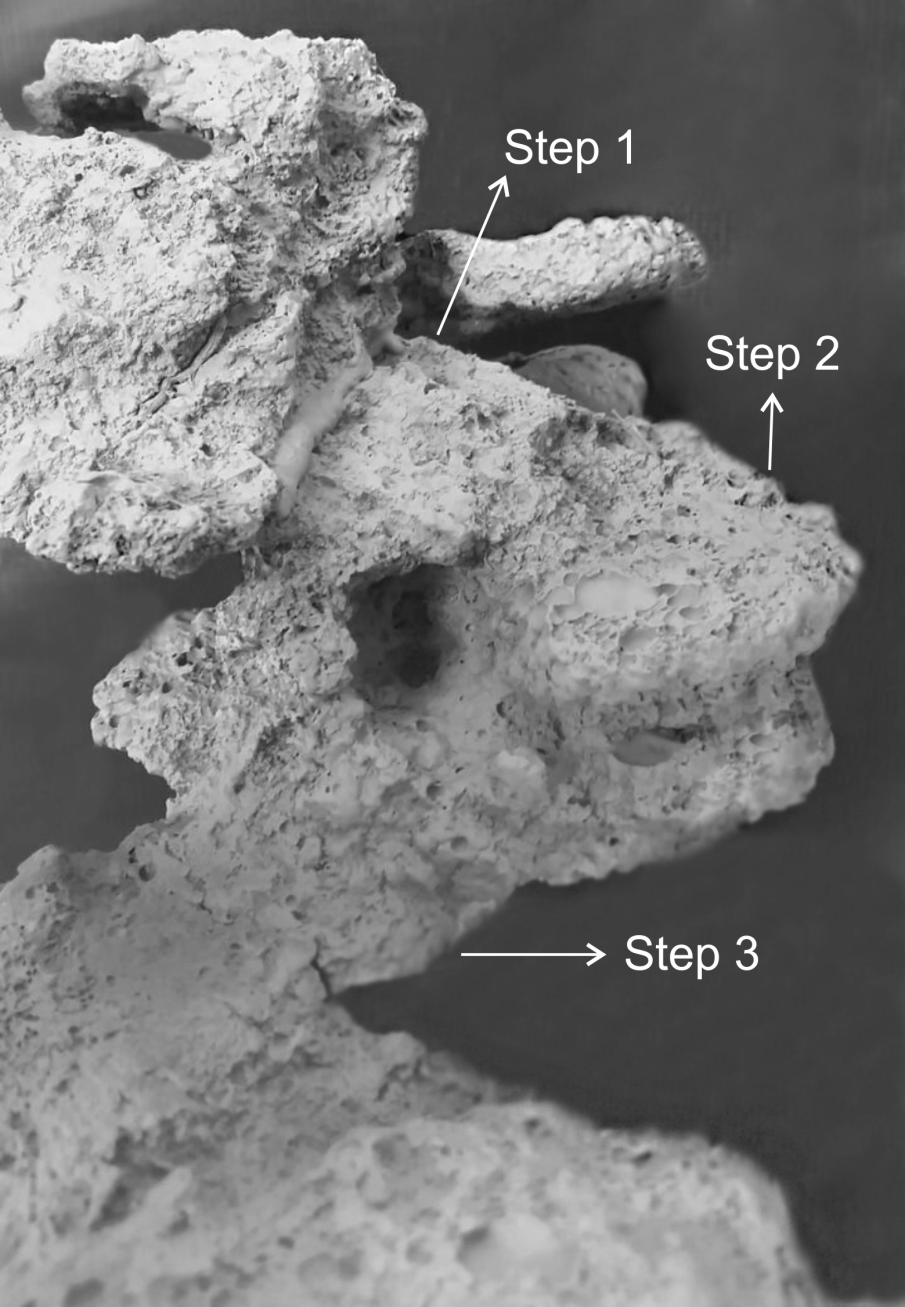
Cement cast of a chamber shaped “in steps”. Chambers built under one another becoming fused, looking like a staircase.

**Fig 7:**
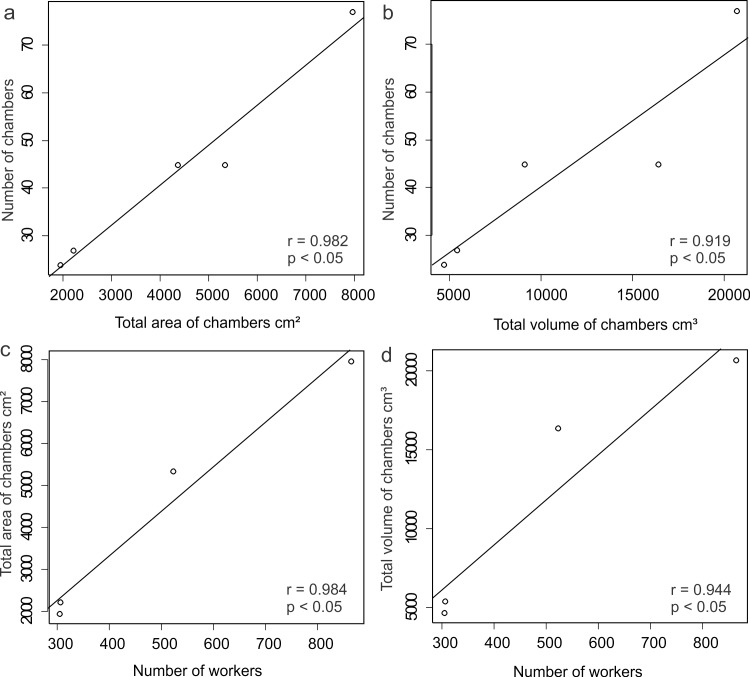
Scatter plot depicting the Pearson’s Correlation tests results. (a) = Pearson’s correlation between total area of chambers and number of chambers; (b) = Pearson’s correlation between total volume of chambers and number of chambers; (c) = Pearson’s correlation between number of workers and total area of chambers; (d) = Pearson’s correlation between number of workers and total volume of chambers; with the respective values of r and p.

**Fig 8:**
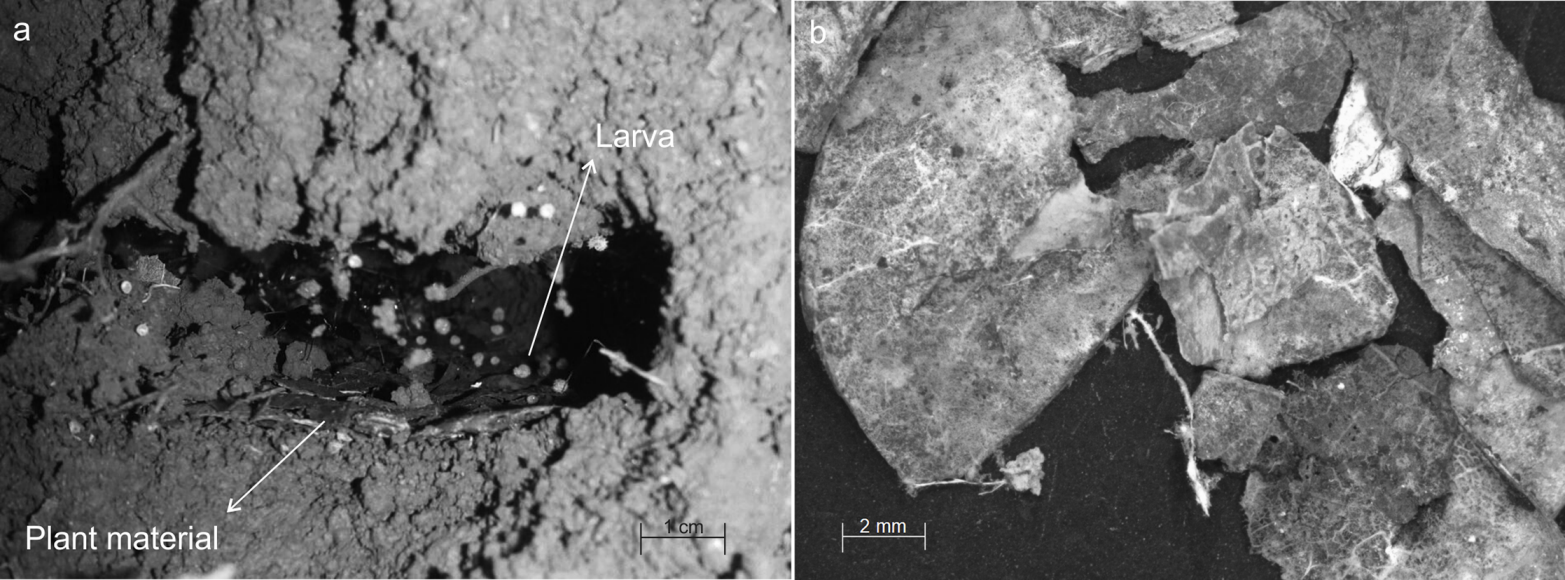
Details of a chamber and fungus present in nests of *Odontomachus chelifer*. (a) = Chamber paved with decaying plant material and presence of immature of the ant *Odontomachus chelifer*; (b) = Detail of the plant material with fungal proliferation.

In general, the chambers had many shapes, oval, circular, “in steps” (Figs 1, 2 and 6), which are probably chambers built under one another so that they became fused, looking like a staircase, or irregular, usually with measures of depth and width larger than height, giving them a flattened appearance (Fig 5B). Due to the presence of many tree roots, some chambers are built following their axes and shapes, so their floor becomes sometimes tilted and not completely horizontal (Fig 5B). Some chambers presented a narrow extension called appendix (Fig 4). Chambers used for garbage storage had smaller dimensions than the others and were generally found in the deepest parts of each shaft (Figs 1,2 and 4). The largest number and volume of chambers were found in the most superficial and middle portions of the nest making them more top-heavy than bottom-heavy (Fig 5B). According to the Pearson’s correlation tests, it is possible to see that the total area and volume of chambers are highly related to the number of chambers (r = 0.982; r = 0.919; p < 0.05) as well as to the number of workers (r = 0.984; r = 0.944; p < 0.05) (Fig 7). In all the nests we found chambers "paved" with pieces of decaying plant material and the presence of a fungus on the floor of some of them (Fig 8).

Apparently, there is no preference for specific chambers for immature allocation, nor were these allocated by stages, since we did not find any pattern of immature distribution in specific chambers and it was possible to find all the stages, eggs, larvae and pupae in the same chamber on more than one occasion. All immature stages were laid directly on the floor or on top of the plant material used for paving the chambers (Fig 8A).

We found several animals cohabiting the nests of *O*. *chelifer*, such as nests of Termitidae termites sharing the same structure of *O*. *chelifer* nests, *Camponotus* sp. ants using the entrance orifices of *O*. *chelifer* nests as entrances for their own nests that were built adjacently to the vertical shafts, as well as specimens of *Pheidole* sp., Gastropoda, Thysanura, Isopoda: Oniscidea, Pseudoscorpiones and Araneae (Table 2).

**Table 2:** Animals cohabiting nests of *Odontomachus chelifer*, other poneromorph species in which these animals have already been described and references for this occurrences.

Animal	Also found in nests of the poneromorph	Reference
Termitidae	*Pchycondyla striata*	[13]
	*Pachycondyla berthoudi*	[26]
	*Pachycondyla foetens*	[27]
	*Plathythyrea schultzei*	[28]
	*Centromyrmex bequaerti*	[29]
	*Ectatomma opaciventre*	[9]
	*Ectatomma brunneum*	[30]
	*Dinoponera quadriceps*	[16]
*Camponotus* sp.	*Ectatomma opaciventre*	[9]
	*Ectatomma brunneum*	[30]
*Pheidole* sp.	*Dinoponera quadriceps*	[18]
	*Dinoponera lucida*	
	*Dinoponera australis*	
Gastropoda	*Dinoponera quadriceps*	[16]
Thysanura	*Dinoponera australis*	[18]
	*Pchycondyla striata*	[13]
Araneae	*Dinoponera australis*	[18]
	*Dinoponera quadriceps*	[16]
Pseudoscorpiones	-	-

## Discussion

Nests of *O*. *chelifer* always presented more than one entrance orifice, covered with plant material from litterfall and near large trees, characteristics that make identification easier, different from what happens in most species of poneromorph ants whose nests present, in general, a single entrance as in cases of *Ectatomma brunneum* Smith 1858 [11], *Ectatomma vizottoi* Almeida Filho 1987 [31] and *O*. *brunneus* [6]. Cases like *Dinoponera gigantea* Perty 1833 whose nests have up to eight entrances [32], *Neoponera marginata* Roger 1861 with up to 11 entrances [33,34] and *P*. *striata* which might present up to 20 entrances [13,34] are considered exceptions. No chimney-like structure or similar occurs in entrance orifices of these nests, unlike in nests of the Ectatomminae *Ectatomma opaciventre* Roger 1861 [9] and *Ectatomma tuberculatum* Olivier 1792 [35] where these structures are commonly present.

Subterranean nests of poneromorph ants are generally considered simple structures consisting of a single vertical shaft with few chambers, and even nests with 16 chambers are considered relatively complex, as is the case of *D*. *quadriceps* [16]. However, nests of *O*. *chelifer* are an exception to the rule, since all of them had more than one vertical shaft and great number of chambers (24 to 77) and connections when compared with other species of the group. Even within the genus *Odontomachus*, these nests deviate from the pattern described so far, since nests of *O*. *brunneus* have only one vertical shaft and maximum of 17 chambers [6]. Fowler [36] studying colonies of *O*. *chelifer* in Paraguay described that they were highly associated with nests of *A*. *sexdens rubropilosa* Linnaeus 1758 and discussed that the minimal excavation effort required by *O*. *chelifer* and the mandibular shape of these ants indicate that they are poor excavators, but we did not find any case of this association despite the presence of colonies of *Atta* Fabricius 1804 in the same area. In addition, the complex structure, great number and area of chambers, tunnels and connections described here indicate that despite their mandible shape these ants might actually excavate very well.

Nests of *O*. *chelifer* have maximum depth, number of chambers, and area per worker increased according to the number of individuals of the colony. Moreover, the total area and volume of chambers are intimately related to the number of chambers, which indicates that nest size grows almost entirely by adding more chambers of a standard size, so that total area and volume remain tightly related to the number of ants. Generally, ants that nest in the soil increase the number of chambers according to the increase in colony’s population, as already described for *E*. *vizottoi* [31], *D*. *quadriceps* [16] and *O*. *brunneus* [6]. Nests of this species have a top-heavy distribution pattern of chambers, as in most ant nests and different from *O*. *brunneus*, whose nests are moderately more bottom-heavy than top-heavy [6].

Nests of *O*. *chelifer* have on average 8.28 ± 1.75 cm^2^ of area per worker, with the ant’s dorsal silhouette without legs and mandibles occupying on average 1.21% of the available area, and 6.50% with legs. Different from nests of *O*. *brunneus* whose area available per worker is approximately 2 cm^2^ regardless of nest size, with ant bodies without legs occupying on average 4.8% of the area available, and 15% with legs [6]. Thus, the value of area per worker found for nests of *O*. *chelifer* is about 4 times larger than the observed in *O*. *brunneus*. Nests of *Pogonomyrmex badius* Latreille 1802 feature on average 1.4 cm^2^ per worker of which worker bodies without legs occupy about 18%, and nests of *Camponotus socius* Roger 1863 have on average 1.1 cm^2^ per worker of which worker bodies without legs occupy a mean of 16% [6].

In all the nests of this species there is at least one chamber with larger dimensions relative to all the others, called “Hall”, as suggested by [31], studying nests of *E*. *vizottoi*, for chambers that have a similar function. This structure acts as a central where there are several tunnels giving access to chambers located both on the same shaft as in different ones. So far, there is no record of Ponerinae species building similar structures to the one described in this study. This system of multiple entrances, chambers and connections, and various sectors of the nest can be related to airflow to maintain its internal conditions, in a pattern similar to that of nests of *Atta* [23,25,37,38,39], in a much smaller scale.

Specific garbage storage chambers were commonly found in the deepest portions of each shaft, as well as in *Ectatomma edentatum* Roger 1863, *E*. *opaciventre*, *E*. *brunneum*, *E*. *vizottoi*, *D*. *quadriceps* and *D*. *australis* [9,11,18,22,30,31]. Something similar happens in nests of *Atta* in which the last chambers also contain garbage and probably have the ability to maintain constant temperature throughout the year because of the production of heat and carbon dioxide by bacterial fermentation [10,14]. However, unlike garbage storage chambers of *Atta* which are usually larger than the superficial ones, in *O*. *chelifer* nests these structures usually have smaller dimensions than the other chambers.

Apparently, *O*. *chelifer* do not allocate their immature neither in specific chambers nor according to the developmental stages in a single chamber, since we did not find any pattern of immature distribution and it was possible to find all the stages, eggs, larvae and pupae in the same chamber on more than one occasion. The same seems to happen in *E*. *brunneum* whose immature are generally found all in a single chamber without any preference as to disposal, depth or volume of chambers [11]. On the other hand, in *P*. *striata* it is often observed a differentiated allocation of pupae relative to larvae and eggs, which may be a derived feature [13]. However, we must consider two things: the first is that allocation can occur dynamically during the day, in search of nest sites with ideal conditions of temperature and humidity [5,13,40]; or the lack of a standard arrangement of immature inside the nest may be due to the disruption caused during excavations which because of the nests complexity could take several days.

Pavement with plant material found in some chambers containing immature may be related to control of temperature and humidity, as suggested by [13] for nests of *P*. *striata* within which there are chambers with immature paved with termite wings. In addition, along with this plant material we sometimes found a fungus developing on the chambers’ floor. The species of this fungus and its function are being investigated, since it is recurrent in all nests. Fungi can affect both workers and immature and if their presence is expected by the ants there must be some mechanism that prevents their proliferation and problems for the population of the colony. Fungus-growing species such as *Atta* and *Acromyrmex* Mayr 1865 for example use antibiotic-producing bacteria that are carried upon the ants’ cuticle to control garden parasites and defend young workers against a fungal entomopathogen [41,42]. Interestingly, some studies have reported the association of *O*. *chelifer* and *D*. *quadriceps* with colonies of the fungus-growing ant *A*. *sexdens rubropilosa* and the presence of a fungus inside *O*. *chelifer* nests might suggest some level of co-evolution between these species [17,36]. However, as mentioned above, we did not observe any case of this association.

Nests of *O*. *chelifer* are cohabitated by several other animals. The use of subterranean ant nests by other animals, mostly arthropods, is well documented in several poneromorph species. As well as in this study, termite nests have also been found associated with nests of many other species, suggesting that this relationship might be widespread in the group [13]. The presence of Pseudoscorpiones in nests of ants of the subfamilies Formicinae and Myrmicinae is well known [43,44] however we believe this is the first record of the occurrence of these animals in nests of poneromorph. According to [14], the use of ant nests by several animals is due to the fact that these structures provide a protective environment, in addition to constant temperature and humidity.

## Conclusions

Nests of *O*. *chelifer* can be considered complex, compared to those currently described for other Ponerinae species, mainly from the same genus. The system of multiple entrances connected directly or indirectly to the Halls, which link various parts of the nest, suggests a complex air circulation system for maintenance of internal conditions.
